# Interplay between endogenous hormones and immune systems in human metapneumovirus pathogenesis and management

**DOI:** 10.3389/fphar.2025.1568828

**Published:** 2025-03-19

**Authors:** Viviana A. Ruiz-Pozo, Santiago Cadena-Ullauri, Rafael Tamayo-Trujillo, Patricia Guevara-Ramírez, Elius Paz-Cruz, Mayra A. Castañeda Cataña, Ana Karina Zambrano

**Affiliations:** ^1^ Universidad UTE, Facultad de Ciencias de la Salud Eugenio Espejo, Centro de Investigación Genética y Genómica, Quito, Ecuador; ^2^ Instituto de Química Biológica de la Facultad de Ciencias Exactas y Naturales (IQUIBICEN). Laboratorio de Estrategias Antivirales, UBA-CONICET, Buenos Aires, Argentina

**Keywords:** healthcare, disease prevention, epidemiology, inflammation, human metapneumovirus, hormones

## Abstract

The present review explores the role of endogenous hormones, such as cortisol, melatonin, thyroid hormones, sex hormones, and insulin, in the modulation of the immune response to a human metapneumovirus (hMPV) infection. hMPV is a respiratory pathogen responsible for severe infections, particularly in vulnerable populations like children and the elderly. The virus triggers inflammatory responses through various molecular processes, including cytokine production and immune signaling pathways. Notably, these processes can be influenced by endocrine factors, such as hormones. Cortisol, through hypothalamic-pituitary-adrenal (HPA) axis activation, modulates inflammation but may contribute to immunosuppression. Melatonin inhibits the NLRP3 inflammasome, reducing lung inflammation. Thyroid hormones regulate immune responses via nuclear factor kappa B (NF-κB) and JAK/STAT pathways, while hypothyroidism may alter infection severity. Sex hormones, particularly estrogens, enhance antiviral immunity, whereas androgens may have variable effects on immune modulation. Insulin influences inflammation through NF-κB suppression, with insulin resistance potentially worsening viral pathogenesis. Therapeutic implications suggest that modulating these hormonal pathways could aid in hMPV management. Strategies such as hormone therapy, glucocorticoid regulation, and nanoparticle-based drug delivery are potential routes of intervention. The aim of the present review is to understand the complex interplay between endogenous hormones and the immune system during an hMPV infection by describing the complex molecular mechanisms associated with these processes.

## Introduction

The human metapneumovirus (hMPV) is a single-stranded, negative-sense RNA virus classified within the genus *Metapneumovirus* (Oketch et al., 2019; Zhang et al., 2024; Piñana et al., 2024). hMPV is considered the primary cause of acute lower respiratory infection in both adults and children worldwide (Zhang et al., 2024). Infection with hMPV induces airway inflammation, coughing, shortness of breath, sore throat, and necrosis of the airway epithelium (Cox et al., 2015). In severe cases, the infection can progress to bronchiolitis or pneumonia (Van Den Bergh et al., 2024).

An increased prevalence and risk of hMPV infection have been reported in children under 2 years of age, the elderly, and individuals with cancer, AIDS, or a history of organ transplantation (Oketch et al., 2019; Zhang et al., 2024). Currently, no approved treatments exist for an hMPV infection; thus, management mainly involves supportive care. However, various potential treatments, including nucleoside analogs and immunoglobulins, are under investigation (Piñana et al., 2024).

hMPV is classified into two major groups (A and B) based on antigenic variation and nucleotide differences in the genes encoding the attachment (G), nucleoprotein (N), and fusion (F) glycoproteins. Additionally, each group is further divided into two subgroups (A1, A2, B1, and B2) according to their Open Reading Frame (ORF) sequences (Oketch et al., 2019).

hMPV is an enveloped virus; thus, its entry into the host cells is mediated by the fusion of the viral and cellular membranes (Cox et al., 2015). Notably, human cells are generally coated with glycoproteins, which are essential for various cellular processes and are often exploited by pathogens for cell entry. In the case of hMPV, the virus utilizes heparan sulfate proteoglycans (HSPGs), expressed in almost all human cell types, to attach to host cells and facilitate viral entry. Furthermore, the hMPV F protein has an arginine-glycine-aspartic acid (RGD) motif that binds to the cell surface integrins, thereby, promoting virus-cell membrane interaction and enhancing viral entry (Van Den Bergh et al., 2024).

The endocrine system influences immunity by releasing molecules such as hormones to modulate the immune response, either by enhancing or weakening it (Sciarra et al., 2023). Conversely, various viruses have developed specific mechanisms to disrupt the proper function of the endocrine system, potentially leading to endocrinopathies (Somasundaram and Gunatilake, 2021). Viruses can damage endocrine cells through direct viral infection or during their replication process using the host machinery, affecting endocrine organs by promoting inflammation and increasing the risks of an autoimmune response through antibody cross-reactivity (Somasundaram and Gunatilake, 2021).

The immune response to an hMPV infection is characterized by the activation of Th17-like cells, which secrete tumor necrosis factor-alpha TNF-α) and interleukin (IL)-6 in the lungs (Soto et al., 2018). Additionally, Th2-like cells are also activated during this process, leading to the secretion of IL-5 and IL-8, which are associated with the JAK/STAT pathway as well as the MAPK and nuclear factor kappa B (NF-κB) pathways, respectively, promoting inflammation (Takatsu et al., 2009; Chan et al., 2017). Moreover, studies in mice have shown that the Toll-like receptor-4 (TLR)-4, which is linked to NF-κB pathways and pro-inflammatory cytokines (Guevara-Ramírez et al., 2023; Ruiz-Pozo et al., 2023; Ruiz-Pozo et al., 2024), plays a role in the immune response to hMPV. A lack of TLR4 was correlated to a decreased immune response, including lower levels of chemokines and cytokines in mice (Velayutham et al., 2013).

On the other hand, hMPV can disrupt inflammatory pathways, including the inhibition of Interferon (IFN) signaling and production via its viral G and SH proteins (Soto et al., 2018; Bao et al., 2008). Similarly, the M2-2 protein of hMPV can inhibit mitochondrial antiviral signaling (MAVS). Since MAVS is required for NF-kB activation, this pathway remains inactive, leading to increased viral replication and cell death (Seth et al., 2005). Furthermore, certain strains of hMPV have evolved mechanisms to evade the type I IFN response because their phosphoprotein P can disrupt Retinoic acid-inducible gene (RIG)-1 activation, thereby inhibiting the inflammatory response (Velayutham et al., 2013).

The aim of the present review is to understand the intricate interaction between endogenous hormones—including cortisol, melatonin, thyroid hormones, sex hormones, and insulin—and the immune response to an hMPV infection by reviewing the complex molecular mechanisms associated with this interplay.

## Endogenous hormones and their influence on hMPV response:

### Cortisol

The hypothalamic-pituitary-adrenal (HPA) axis is a neuroendocrine system that connects the central nervous system with peripheral tissues. It regulates homeostasis, under conditions of physiological and pathological stress, such as viral infections (Sunstrum and Inoue, 2019; Weng et al., 2023). In hMPV infection, activation of the HPA axis and subsequent cortisol release attempt to prevent tissue damage caused by uncontrolled inflammation. Cortisol, a glucocorticoid with immunomodulatory properties, plays a central role in regulating the inflammatory response and modulating pro-inflammatory cytokines (Herman et al., 2016).

During hMPV infection, the release of pro-inflammatory cytokines activates the HPA axis as part of the immune response. This activation begins in the central nervous system, where the hypothalamus, in response to inflammatory signals, stimulates the release of corticotropin-releasing hormone (CRH). In the early stages of infection, innate cytokines such as IL-1, IL-6, and TNF- α induce CRH secretion by neurons in the paraventricular nucleus (PVN) of the hypothalamus. These neurons project their endings to the median eminence (ME), where CRH is released into the portal-pituitary circulation, promoting activation of the HPA axis and subsequent endocrine response (Somasundaram and Gunatilake, 2021; Silverman et al., 2005).

CRH binds to CRH-R1 receptors on anterior pituitary corticotropic cells, triggering the synthesis and release of adrenocorticotropic hormone (ACTH). ACTH travels through the circulation to the adrenal glands, where it acts on MC2-R type 2 melanocortin receptors in the adrenal cortex, inducing the production of glucocorticoids such as cortisol (Cox et al., 2015). Cortisol regulates excessive inflammation through its interaction with glucocorticoid receptors (GR) in the cytosol of target cells. Binding cortisol to GR causes translocation of the receptor-ligand complex to the nucleus, where it regulates the transcription of genes involved in inflammation and antiviral response, modulating transcription factors such as NF-κB and AP-1 (Borghetti et al., 2009; Somasundaram and Gunatilake, 2021).

In advanced stages of hMPV infection, adaptive cytokines such as TNF-α, INF-γ, and IL-2 maintain active glucocorticoid production through the HPA axis to control systemic inflammation (Silverman et al., 2005; Borghetti et al., 2009; Gjerstad et al., 2018). However, negative feedback from glucocorticoids regulates this response by inhibiting CRH and ACTH release, desensitizing CRH-R1 receptors, and limiting axis activity. The hippocampus, with a high density of glucocorticoid receptors, also contributes to this regulation of HPA, protecting the body from excessive glucocorticoid exposure and reducing the risk of catabolic and immunosuppressive effects (Gjerstad et al., 2018).

Although respiratory symptoms are the main manifestations of hMPV, studies have identified its presence in cerebrospinal fluid (CSF), indicating that the virus can spread from the lungs to the central nervous system (CNS), causing complications such as encephalitis (Jeannet et al., 2017; Schildgen et al., 2005; Kalergis et al., 2023). This behavior could be linked to generalized inflammatory activation and HPA axis response, highlighting the need to understand how interactions between the immune system, glucocorticoids, and viral shedding contribute to the neurological manifestations observed in severe hMPV infections (Bohmwald et al., 2018).

#### Melatonin

The pineal gland’s primary hormone, melatonin, is essential for both human and animal pharmacological and pathophysiological states (Hosseinzadeh et al., 2016; Daryani et al., 2018). This hormone has a variety of functions, including immunoregulatory, anti-inflammatory, anti-excitatory, sleep-initiating, and antioxidant (Bahrampour Juybari et al., 2020).

The NLRP3 inflammasome is an oligomeric complex essential for host defense against viruses, as it increases the release of IL-1β and IL-18 and induces pyroptosis. This inflammasome recognizes pathogen-associated molecular patterns (PAMPs) and damage-associated molecular patterns (DAMPs) generated during viral replication, leading to antiviral immune responses and viral elimination (Lupfer et al., 2020).

Interestingly, Le et al. (2019) found that the NLRP3 inflammasome has a detrimental effect during hMPV infection. Its inhibition protected mice from mortality and reduced weight loss and inflammation without affecting viral replication. This negative effect was attributed to IL-1β production, as IL-1β-deficient mice exhibited less mortality, weight loss, and inflammation compared to wild-type mice. The research group also observed that activation of the NLRP3 inflammasome by the hMPV SH protein promotes IL-1β maturation, exacerbating hMPV-induced inflammation. Therefore, blocking IL-1β production with NLRP3 inflammasome inhibitors could be a novel therapeutic and preventive strategy for hMPV infection (Le et al., 2019).

In this context, Zhang et al. (2016) demonstrated that melatonin is a potent NLRP3 inflammasome inhibitor in an LPS-induced acute lung injury (ALI) mouse model. This beneficial effect of melatonin ameliorates lung damage and reduces neutrophil and macrophage influx into the lungs (Zhang et al., 2016).

Similarly, Wu et al. (2020) suggested that melatonin could be a promising therapeutic drug for inflammatory airway diseases. They demonstrated that an endogenous TLR2-melatonin feedback loop regulates NLRP3 inflammasome activation in allergic airway inflammation. Suppression of melatonin synthesis by TLR2 activation results in the loss of its inhibitory effect on TLR2 signaling (Wu et al., 2020).

#### Thyroid hormones

Triiodothyronine (T3) and thyroxine (T4) are the main thyroid hormones that regulate metabolism, development, cardiac frequency, and immune responses (Brent, 2012). Their production is regulated by the hypothalamic-pituitary-thyroid axis, which operates as a feedback loop to maintain hormonal balance. The hypothalamus secretes thyrotropin-releasing hormone (TRH), promoting the anterior pituitary to release thyrotropin-stimulating hormone (TSH). TSH acts on the thyroid gland, promoting the production of T4, which is then converted into the active form, T3, by enzymes called deiodinases (Astapova et al., 2011; Feldt-Rasmussen et al., 2021). Thyroid hormones are transported across the cell membrane by proteins such as monocarboxylate transporter 8 (MCT8) to enter the cells (Mebis and van den Berghe, 2009). Once inside, thyroid hormones can act by binding to different molecules located on the plasma membrane, mediated by binding to thyroid nuclear receptors (Lasa et al., 2022).

Thyroid hormones play an essential role in interacting with molecular pathways like the immune response to respiratory viruses (Lasa et al., 2022). They modulate the activity of various immune cells, such as neutrophils, macrophages, natural killer cells, dendritic cells, and B and T lymphocytes, while also influencing key inflammatory pathways (Rubingh et al., 2020). For example, thyroid hormones influence the transcription of genes involved in inflammatory pathways, such as the NF-kB pathway, p38MAPK signaling pathway, and JAK/STAT signal cascade (Lasa et al., 2022; Wenzek et al., 2022). During acute infection, pro-inflammatory cytokines can suppress the production and activity of T4 and T3, resulting in non-thyroidal illness syndrome (NTIS) (Astapova et al., 2011; Aranda, 2025). Additionally, studies demonstrated that the function of macrophages and cytokine production could be affected by thyroid hormones (Wenzek et al., 2022).

In the case of hMPV infections, thyroid hormones can influence immune responses and the progression of the disease. Research has investigated the immune response to hMPV under hypothyroxinemia (Hpx) in gestated mice and found that Hpx females had a more effective immune response in reducing viral loads compared to Hpx males. These findings suggest that disruptions in thyroid hormone levels during hMPV infections may compromise protective immune mechanisms, potentially exacerbating symptoms and delaying recovery (Funes et al., 2022).

#### Sex hormones

Endogenous sex hormones, including estrogens, progesterone, and androgens, are synthesized mainly in the gonads and the adrenal cortex (Delchev and Georgieva, 2018). The regulation of these hormones is provided by the hypothalamic-pituitary-gonadal (HPG) axis that promotes the activation of Gonadotropin-releasing hormone (GnRH) to direct the pituitary gland to release Luteinizing hormone (LH) and follicle-stimulating hormone (FSH) (Hu et al., 2010). LH and FSH hormones act on the gonads and synthesize sex hormones such as estrogens (e.g., estradiol), progesterone, and androgens (Lomauro and Aliverti, 2021; Acevedo-Rodriguez et al., 2018).

In the process of respiratory virus infection in the host, sex hormones influence the immune response through different molecular pathways. Estrogens bind to estrogen receptors (ER), ERα, and ERβ on immune cells such as B and T lymphocytes, macrophages, and dendritic cells. In addition, ERs act on Calcium regulation by inducing the activation of some metabolic pathways such as ERK/MAPK and PI3K/AKT through proteins such as calmodulin and Calcium-dependent kinases and JAK-STAT activated by cytokines; metabolic processes that promote type I and III IFN responses involved in antiviral defense (Ghosh and Klein, 2017; Hu et al., 2023).

Androgens, acting through the androgen receptor (AR), form an androgen-AR complex to modulate the transcription of target genes and activate the calcium cascade to promote the ERK, AKT, and MAPK signaling pathways involved in virus defense. Thus, exposure of monocytes and macrophages to androgens triggers the decrease of pro-inflammatory responses by regulating cytokines and IFNs. For example, research mentions that alveolar macrophages enhance IL-4-induced M2 polarization of macrophages. However, in some cases, inflammatory processes are enhanced by androgens, which may be due to the different types of macrophages in the tissues and may reduce the efficacy of viral clearance (Becerra-Diaz et al., 2020).

Progesterone is another sex hormone involved in the innate antiviral response. Studies in mice infected with a respiratory virus identified an increased level of progesterone, suggesting that this hormone promotes the immune response by stimulating progesterone receptors (PGRs) (Kadel and Kovats, 2018), which activate the SRC tyrosine kinase. This activation induces phosphorylation of interferon regulatory factor 3 (IRF3) at tyrosine residue 107 (Y107), promoting the activation of antiviral genes and triggering the immune response against infection (Su et al., 2022). In addition, IRF3 induces the transcription of genes encoding for type I and type III IFN, promoting this immune pathway as an antiviral defense (Mogensen, 2019).

Some respiratory viruses could be associated with host proteases to infect cells, in the case of the respiratory virus hMPV, it has been observed that the transmembrane serine protease subtype 2 (TMPRSS2) activates the fusion protein for viral entry. Moreover, this protease would be regulated by androgenic hormones through its receptors (Böttcher-Friebertshäuser, 2018). Immunostaining studies of human respiratory tract tissues such as bronchi and alveoli have observed the expression of the TMPRSS2 protein as a factor that can favor proteolytic activation and, consequently, the propagation of hMPV (Wettstein et al., 2022).

#### Insulin and insulin resistance

Insulin is the main anabolic hormone that regulates carbohydrates, lipids, and protein metabolism. Insulin is synthesized in pancreatic beta cells where the insulin gene encodes a single chain prohormone, which is modified in the Endoplasmic Reticulum and Golgi apparatus to generate a mature insulin hormone. These mature molecules are secreted after high glucose plasma concentrations and promote the uptake of glucose in a GLUT transporter-dependent manner (Baumgard et al., 2016). However, this insulin production and signaling could be altered under pathological conditions. The insulin effect is through the stimulation of nitric oxide production and suppression of pro-inflammatory transcription factors like NF-κB, and subsequently, the reduction of pro-inflammatory cytokines (IL-1β, IL-6, TNF-α) (Dandona et al., 2006; Dandona et al., 2009). Insulin also suppresses the gene expression of TLRs, which leads to a less inflammatory response (Dandona et al., 2009; Ghanim et al., 2008). Therefore, insulin treatment in hMPV infections could improve the symptoms and prevent energy metabolic disorders. Nano-delivery strategies (Mendes et al., 2022) could be useful to allow enhanced insulin signaling in infected airway epithelial cells.

However, insulin treatment in viral infections must be closely monitored due to the possibility of hypoglycemic events, which could worsen the individual’s health status (Ursino et al., 2024). Therefore, co-treatment with adjuvants that prevent hypoglycemic events could be evaluated in animal models to optimize insulin treatment against hMPV infections.

Insulin Resistance (IR) is a complex condition that leads to an impaired sensitivity to insulin in peripheral tissues. This condition is characterized by hyperglycemia, hyperinsulinemia, abnormal lipid accumulation, altered cellular glucose uptake, and increased lipid oxidation in adipocytes (Zhao et al., 2023). The main cause of IR is linked to an inflammatory process promoted by overnutrition, which induces the production of pro-inflammatory cytokines like IL-1β, IL-6, TNF-α, adipokines, and chemokines (Rehman and Akash, 2016). The cytokines overproduction leads to the activation of the TLRs, which also induces several pro-inflammatory transcription factors and signaling cascades NF-κB, c-Jun N-terminal kinase (JNK), inhibitor of kappa B kinase-β (IKKβ)) (Rehman and Akash, 2016; Tilg and Moschen, 2008). Hence, chronic overnutrition triggers chronic inflammation, which causes deleterious effects in beta-cell pancreatic islets, promoting insulin depletion and worsening the inflammatory state (Tilg and Moschen, 2008; Yan et al., 2024). Therefore, the energy metabolism disorders produced by IR could worsen protein turnover and facilitate viral infection.

An inflammatory process due to viral infections also requires the interaction of viral particles with the TLRs. This process also promotes the cellular activation of the transcription factor NF-κB, which leads to the production of pro-inflammatory cytokines (IL-1β, IL-6, TNF-α); this process has been described in the airway epithelial cells infected with hMPV (Kolli et al., 2012). The role of insulin as an anti-inflammatory effector has been described previously (Sun et al., 2014). Therefore, insulin could be assessed as an anti-inflammatory agent against hMPV infection.

### Immunosenescence and hormones

Immunosenescence refers to the age-related decline in immune function, this process is further exacerbated by hormonal imbalances associated with aging (Arlt and Hewison, 2004). Cortisol release increases with aging, potentially weakening the immune response following an infection (Stamou et al., 2023). Conversely, melatonin, thyroid hormones, and sex hormones production decreases over time, these processes have been linked to a diminished and dysregulated immune function (Yoo et al., 2016; De Vito et al., 2011; Bupp et al., 2018). Additionally, aging disrupts insulin-related processes, often resulting in hyperinsulinemia and insulin resistance, which can compromise immune function and promote chronic inflammation (Shimi et al., 2024).

### Therapeutic implications

The antiviral therapy used to control hMPV infection is still being tested. It employs ribavirin, immunoglobulin, fusion inhibitors, and interfering ribonucleic acids to treat and control hMPV (Pasikhova et al., 2018; Wyde et al., 2003; Shahani et al., 2017; Panda et al., 2014; Darniot et al., 2012; Shafagati and Williams, 2018). Thus, there is currently no approved therapeutic or preventive medication for the management of hMPV infection. Vaccine development is difficult without a reliable animal model, making preclinical studies and effectiveness testing even harder (Wyde et al., 2003; Stepanova et al., 2020).

In viral infections, glucocorticoids play a role in regulating immune responses by reducing pro-inflammatory cytokines and promoting anti-inflammatory mediators through glucocorticoid receptors. Specifically, in hMPV infections, the inflammation can be managed by glucocorticoid receptor modulators (Liberman et al., 2018; Li et al., 2021). However, researchers have found mixed results when using glucocorticoids and ribavirin in severe viral illnesses like hMPV infection (Hamelin et al., 2006). A study involving combination therapy with intravenous ribavirin and immunoglobulin has shown potential to manage hMPV pneumonia, but further studies are required (Bonney et al., 2009).

**FIGURE 1:**
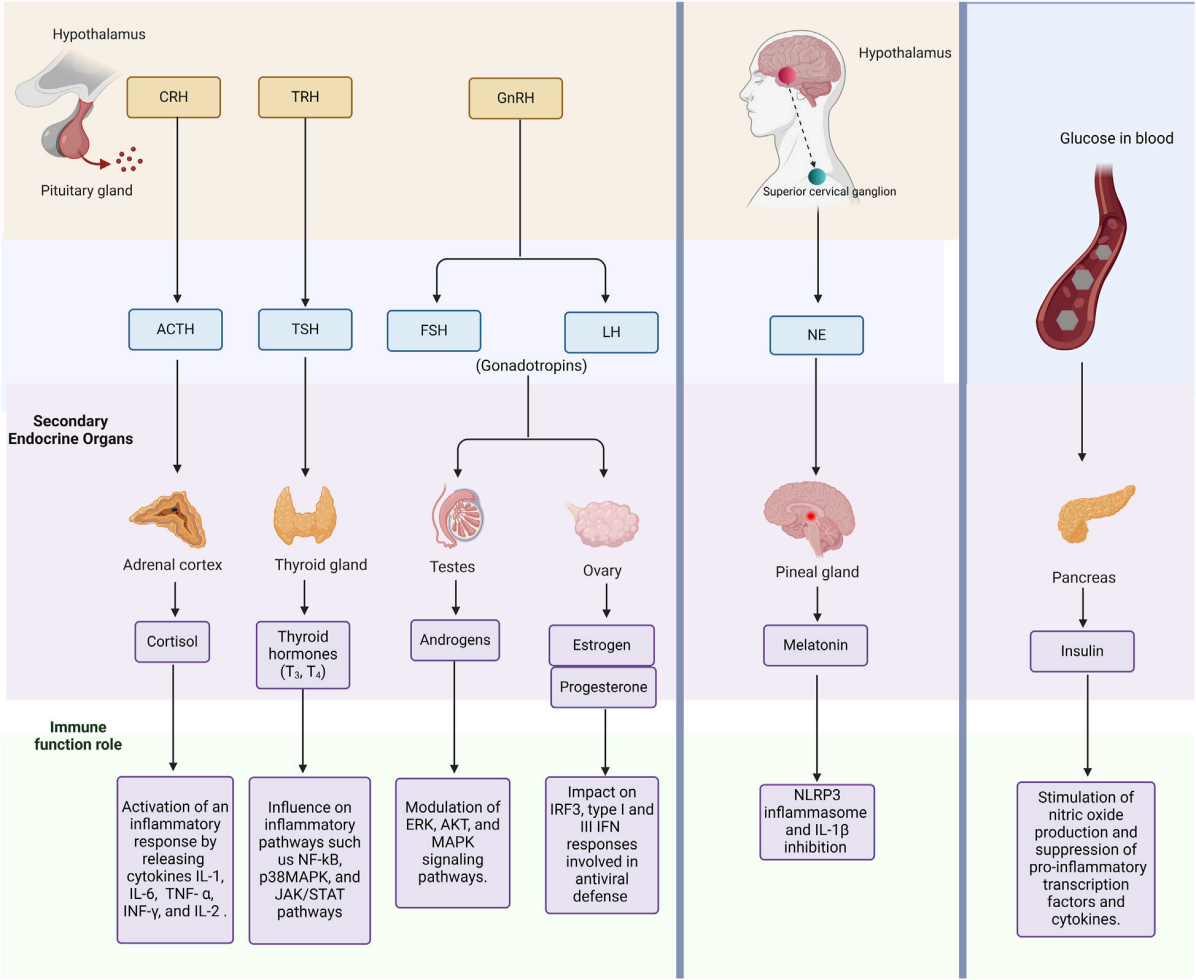
Overview of Endogenous Hormonal Pathways and Their Interaction with the Immune System. This figure highlights key endogenous hormones and signaling pathways. In addition, it describes how endocrine signaling modulates immune responses, including inflammation and immune cell activation. Abbreviations: CRH, corticotropin-releasing hormone; TRH, thyrotropin-releasing hormone; GnRH, Gonadotropin-releasing hormone; ACTH, adrenocorticotropic hormone; TSH, thyrotropin-stimulating hormone; FSH, follicle-stimulating hormone; LH, Luteinizing hormone; NE, Noradrenaline; IL-1, Interleukin 1; IL-6, Interleukin 6; TNF- α, Tumour Necrosis Factor alpha; interferon gamma, INF-γ; and IL-2, Interleukin 2; NF-kB, nuclear factor-kappa B; MAPK, mitogen-activated protein kinase; ERK, Extracellular signal-regulated kinase; AKT, Protein kinase B; IRF3, interferon regulatory factor 3, IFN, Interferon; and NLRP3, NOD-like receptor protein 3.

Hormonal signaling pathways present promising targets for anti-inflammatory strategies in managing hMPV infections (Kino and Chrousos, 2007). This relationship is illustrated in Figure 1. For instance, estrogen signaling has been shown to enhance antiviral responses by upregulating interferon-stimulated genes (ISGs) (Uche and Guerrero-Plata, 2018), while androgen signaling can potentially suppress these effects (Zou et al., 2022). By modulating these pathways through selective estrogen receptor modulators (SERMs) or AR antagonists, it may be possible to develop more targeted therapeutic interventions (Li et al., 2022; Abramenko et al., 2021). Similarly, melatonin’s anti-inflammatory and antioxidant properties can be harnessed for reducing lung injury and oxidative stress, offering a multi-faceted approach to treating hMPV (Silvestri and Rossi, 2013; Boga et al., 2012).

Nanoparticles, due to their diminutive size, tunable properties, and capacity for information transport, enhance the stability, efficacy, and precision of therapeutic delivery and represent a promising approach to improve the delivery of hormones and antiviral molecules, potentially resulting in more effective and personalized treatments for hMPV (Singh et al., 2017). (Darniot et al., 2012; Wen and Williams, 2015). Moreover, their antiviral and biomimetic properties strengthen treatment effectiveness, and when combined with other antiviral methods, they may further improve clinical outcomes (Castañeda Cataña et al., 2025; Castañeda Cataña et al., 2024). According to other studies, nanoparticles loaded with IFN are a new choice because they are straightforward to use (inhaled dry powder) and can be delivered by the nose to children and older adults without issues (Ramos et al., 2024).

## Conclusion

In conclusion, hMPV infection is a complex process involving intricate interactions between the virus and the host, including the modulation of the immune response by endogenous hormones through various molecular pathways. Future research should focus on personalized medicine approaches and the study of hormone-based interventions in viral respiratory diseases.
